# Prevalence, Antimicrobial Resistance, and Virulence Potential of *Staphylococcus aureus* in Donkeys from Nigeria

**DOI:** 10.3390/antibiotics14050453

**Published:** 2025-04-29

**Authors:** Onyinye Josephine Okorie-Kanu, Madubuike Umunna Anyanwu, Obichukwu Chisom Nwobi, Regina Yaya Tambe-Ebot, Nkechi Harriet Ikenna-Ezeh, Chukwuemeka Calistus Okolo, Lynda Onyinyechi Obodoechi, Patience Chinasa Ugwu, Ifeyinwa Riona Okosi, Ishmael Festus Jaja, James Wabwire Oguttu

**Affiliations:** 1 Department of Veterinary Public Health and Preventive Medicine, University of Nigeria, Nsukka 402001, Nigeria; onyinye.okoro@unn.edu.ng (O.J.O.-K.); obichukwu.nwobi@unn.edu.ng (O.C.N.); yaya.nsor.235961@unn.edu.ng (R.Y.T.-E.); lynda.majesty-alukagberie@unn.edu.ng (L.O.O.); 2 Department of Veterinary Microbiology and Immunology, University of Nigeria, Nsukka 402001, Nigeria; nkechi.ikenna-ezeh@unn.edu.ng; 3 Department of Veterinary Medicine, University of Nigeria, Nsukka 400001, Nigeria; chukwuemeka.okolo@unn.edu.ng; 4 Department of Animal Health and Production, Faculty of Veterinary Medicine, University of Nigeria, Nsukka 402001, Nigeria; chinasa.ugwu@unn.edu.ng; 5 National Veterinary Research Institute, Vom 930001, Nigeria; ify.okosi@nvri.gov.ng; 6 Department of Livestock and Pasture Science, University of Fort Hare, Alice 5700, South Africa; 7 SAMRC Microbial Water Quality Monitoring Centre, University of Fort Hare, Alice 5700, South Africa; 8 Department of Agriculture and Animal Health, University of South Africa, Roodepoort, Johannesburg 1710, South Africa; joguttu@unisa.ac.za

**Keywords:** antimicrobial resistance, donkeys, methicillin resistance, staphylococci, virulence potentials

## Abstract

Background: Animal-associated antimicrobial-resistant staphylococci pose a One Health concern, as they can spread into the environment and cause serious infections. Yet, donkeys in Nigeria have been largely overlooked as potential reservoirs of these pathogens. Aim/Objectives: To isolate *Staphylococcus aureus* from donkeys in Obollo-Afor, southeast Nigeria, assess their antimicrobial resistance profiles, and evaluate their virulence potential. Materials and Methods: Staphylococci were isolated from the nasal swabs of 250 donkeys, using mannitol salt agar, confirmed biochemically, with *Staphylococcus aureus* identified via a latex agglutination test and mass spectrometry. The resistance profiles of the isolates, including in regard to methicillin, inducible clindamycin, and β-lactamase production, were determined using disc diffusion, while vancomycin resistance was assessed through the use of agar dilution. The virulence factors were evaluated phenotypically. Results: Of the 250 samples, 11 (4.4%) contained *S*. *aureus* and 239 (95.6%) grew other *Staphylococcus* species. The resistance rates of the 11 *S*. *aureus* isolates to gentamicin, penicillin, tigecycline, cefoxitin, linezolid, and chloramphenicol were 45.5%, 66.7%, 54.5%, 27.3%, 36.4%, and 18.1%, respectively. The phenotypic methicillin-resistant *S*. *aureus* prevalence was 1.2%. Additionally, 23.5% of the *S*. *aureus* isolates were multidrug resistant, with a mean antibiotic resistance index of 0.25. All the *S*. *aureus* isolates exhibited virulence factors like clumping factor expression, catalase, caseinase, lecithinase, and gelatinase activity, while the occurrence of haemagglutinin, biofilm, pellicle, and hemolysin occurred in 27.3%, 54.5%, 36.4%, 72.2%, respectively. Conclusion: Although a small percentage of donkeys in Nigeria may harbor *S*. *aureus*, these animals are potentially spreading antimicrobial resistance, including multidrug and methicillin resistance, to humans and the environment.

## 1. Introduction

*Staphylococcus aureus* is a commensal bacterium found on the skin and mucosal surfaces of humans and animals, including donkeys. However, it can also act as an opportunistic pathogen, causing infections ranging from mild skin conditions to severe diseases, such as necrotizing fasciitis, endocarditis, osteomyelitis, and septicemia [1,2]. The transmission of *S*. *aureus* occurs through direct contact with colonized hosts or contaminated environments, facilitating its spread across ecological niches [3]. The virulence of *S*. *aureus* is linked to structural and extracellular factors, including biofilm formation, which enhances antimicrobial resistance and persistence; hemagglutinins, which facilitate adherence to host tissues; and hydrolytic enzymes, such as coagulase, hemolysins, and lipases, which contribute to immune evasion and tissue invasion [4,5]. Staphylococcal strains that possess and express virulence factors pose a more significant threat than those lacking them. Therefore, a phenotypic assessment of the virulence factors is essential, as the mere presence of a virulence-associated gene does not guarantee its expression, particularly when multiple genes are required for a virulence trait to manifest [6].

The emergence of antimicrobial resistance among *S*. *aureus* strains, particularly resistance to critically important antibiotics (CIAs), such as oxacillin (methicillin), vancomycin, linezolid, clindamycin, and tigecycline, presents a significant public health threat. *S*. *aureus* isolates that are resistant to these CIAs are potential “superbugs”, often responsible for severe, difficult-to-treat multidrug-resistant infections, leading to increased healthcare costs and morbidity and mortality, due to limited treatment options [7]. Between 2019 and 2021, *S*. *aureus* was responsible for approximately 130,000 to 1 million deaths out of 114 million to 127 million deaths attributable to bacterial antimicrobial resistance [8,9,10]. It is one of the highly resistant ESKAPE (*Enterococcus faecium/faecalis*, *Staphylococcus aureus*, *Klebsiella pneumoniae*, *Acinetobacter baumannii*, *Pseudomonas aeruginosa*, and *Enterobacter cloacae*) organisms, which are responsible for the high morbimortality associated with difficult-to-treat community- and hospital-associated infections, including pneumonia and blood stream infections [10,11]. Indeed, *S*. *aureus* is among the high-priority bacteria designated for surveillance by the World Health Organization (WHO), due to its role in multidrug-resistant infections [12]. In Nigeria, weak regulations on antimicrobial use in veterinary medicine have led to the widespread use of empirical antibiotic treatment without susceptibility testing, further driving resistance development. Furthermore, the prophylactic and empirical use of antibiotics in donkeys, as in horses, without conducting antibiotic sensitivity testing, is permitted globally [13]. Donkeys, through their exposure to antibiotics and environmental reservoirs of resistant bacteria, may harbor and disseminate multidrug-resistant staphylococci.

Nigeria has an estimated donkey population exceeding one million [14], primarily concentrated in the northern regions, where they are used for various purposes, including water drawing, threshing, farm cultivation, transportation, and meat, milk, and hide production (for gelatin manufacturing) [15,16,17]. They are also utilized for companionship, therapeutic activities, and ecotourism [18]. Despite government regulations prohibiting the donkey meat trade, its consumption is increasing, constituting a notable portion of total meat consumption in Nigeria [19]. Donkeys colonized by pathogenic antimicrobial-resistant staphylococci pose significant public health and food safety concerns. These animals can serve as reservoirs for bacteria, facilitating their transmission to humans through direct contact, meat consumption, or exposure to contaminated environments. *S. aureus* is a cause of particular concern due to its ability to produce heat-stable enterotoxins, which remain active despite the use of standard cooking temperatures, and can cause severe foodborne illnesses, characterized by nausea, vomiting, and diarrhea [20]. In Nigeria, individuals interacting with donkeys, such as farmers, transporters, caretakers, vendors, and butchers, rarely use protective equipment or practice proper hand hygiene, increasing the risk of bacterial transmission. Additionally, donkey meat processing, particularly in southeastern Nigeria, often occurs in suboptimal hygienic conditions, heightening the risk of microbial contamination. As a result, donkeys can introduce staphylococci into Nigerian food-processing environments, potentially leading to large-scale foodborne disease outbreaks that impact both individuals and communities. The ability of staphylococci to persist in various food matrices, including dairy, meat, and ready-to-eat products, further underscores the necessity for routine *S*. *aureus* surveillance in food animals to prevent contamination and ensure public health safety. Given that staphylococci naturally inhabit donkeys’ skin and mucosal surfaces, potential donkey meat contamination sources include colonized slaughterhouse personnel, unhygienic equipment, and contaminated water used during meat processing [3]. Consequently, donkeys in Nigeria may serve as reservoirs for zoonotic pathogens, including antimicrobial-resistant *S*. *aureus*, raising significant One Health concerns. 

Despite the zoonotic potential of *Staphylococcus* species in donkeys, research on their occurrence, antimicrobial resistance, and virulence characteristics remains limited. Studies from Ethiopia [21], Egypt [22], Tunisia [23,24], Portugal [18], Italy [25,26], and the USA [27] have documented staphylococcal colonization in donkeys, but similar investigations in Nigeria are scarce. Understanding the prevalence and resistance profiles of these organisms in donkeys is critical for mitigating public health risks. This study aimed to isolate *S*. *aureus* from donkeys at a major livestock market in Obollo-Afor, southeast Nigeria, characterize their antimicrobial resistance profiles, and evaluate their virulence potential.

## 2. Results

### 2.1. Occurrence of S. aureus in Donkeys

Out of 250 nasal swabs processed, 11 (4.4%, 95% CI: 1.86% - 6.94%) grew non-repetitive mannitol-fermenting *S. aureus*, while 239 (95.6%, 95% CI: 93.06% - 98.14%) yielded other *Staphylococcus* species, which comprised 199 that grew non-*aureus* mannitol-fermenting strains and 40 that grew mannitol non-fermenting staphylococci (Table 1). There was a significant association (*p* < 0.001) between the donkeys’ colonization rate and the *Staphylococcus* species.

### 2.2. Antimicrobial Resistance Profile of the Isolates

The antimicrobial resistance profile of the 11 *S*. *aureus* isolates against 10 antimicrobial agents (Table 2) revealed that all the isolates were resistant to at least two antimicrobial agents, with the following resistance rates: penicillin (66.7%; 95% CI: 38.85–94.55%), cefoxitin (27.3%; 95% CI: 0.97–53.63%), tigecycline (54.5%; 95% CI: 25.07–83.93%), gentamicin (45.5%; 95% CI: 7.97–64.83%), linezolid (36.4%; 95% CI: 7.97–64.83%), and chloramphenicol (18.1%; 95% CI: -4.65–40.85%) (Table 2). There were significant differences in the susceptibility distribution of doxycycline (*p* < 0.001) and chloramphenicol (*p* < 0.01). The sample prevalence of methicillin-resistant *S*. *aureus* was 1.2% (3/250), while that of methicillin-susceptible *S. aureus* (MSSA) was 3.2% (8/250). Notably, none of the isolates exhibited resistance to clindamycin, erythromycin, or vancomycin.

Among the 11 *S. aureus* isolates, 54.5% were resistant to two antimicrobial agents, 18.2% to three, 18.2% to four, and 9.1% to five (Table 3). 

The 11 resistant isolates exhibited seven distinct multi-resistance patterns (i.e., resistance to two or more antimicrobial agents), with PEN–LZD being the most common (*n* = 3) (Table 4). Six (45.5%), including the MRSA strains of the 11 *S. aureus* isolates, were classified as multidrug-resistant (MDR). The mean multiple antibiotic resistance index (MARI) of the 11 resistant isolates was 0.25, ranging from 0.2 to 0.5 (Table 4).

### 2.3. Virulence Potential of S. aureus Isolates

**Figure 1:**
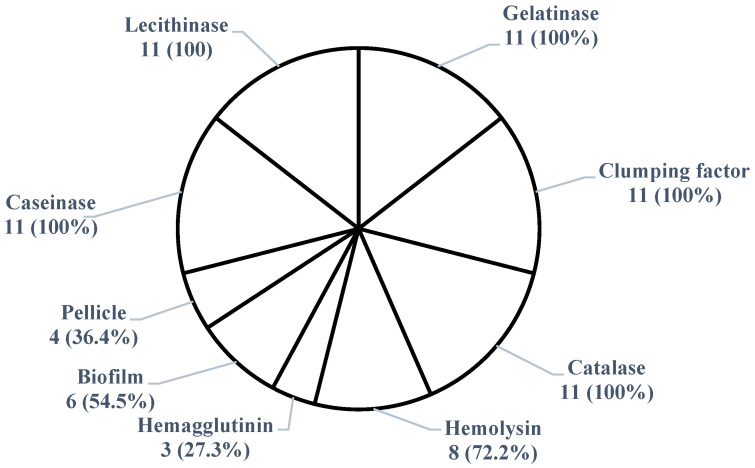
Frequency of phenotypic virulence factors expressed by *Staphylococcus aureus* isolates (*n* = 11) from donkeys in Obollo-Afor, southeast Nigeria.

**Figure 2:**
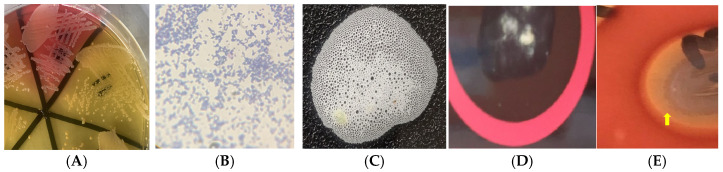

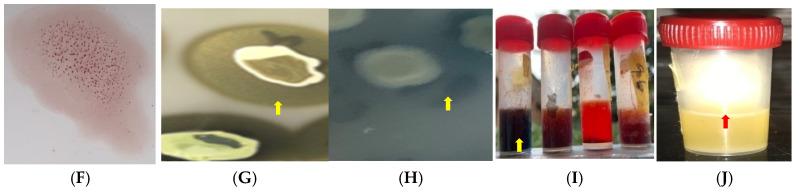
Phenotypic and virulence features of *Staphylococcus aureus* from donkeys in Nigeria: (**A**) mannitol-fermenting (yellowish) staphylococcal isolates on mannitol salt agar; (**B**) Gram-positive cocci in irregular clusters; (**C**) bubbling indicating catalase activity; (**D**) clumping on Staphytect^®^ latex indicating clumping factor expression; (**E**) hemolysis indicated by clear halo around isolate on blood agar; (**F**) hemagglutination of chicken red blood cells; (**G**) gelatin hydrolysis indicated by halo around isolate on gelatin agar; (**H**) lecithinase activity indicated by halo around isolate on egg-yolk agar; (**I**) biofilm expression indicated by blackening of Congo red broth; and (**J**) pellicle formation in tryptic broth. Arrow indicates positive result.

All 11 *S*. *aureus* isolates exhibited virulence factors. Among them, 100% showed catalase, lecithinase, gelatinase, caseinase activities, and clumping factor expression, 72.2% displayed hemolysis, 54.5% developed a biofilm, 36.4% exhibited pellicle, and 27.3% demonstrated hemagglutination (Figure 1 and Figure 2). The 11 *S*. *aureus* strains expressed a combination of nine of the tested virulence factors. There was no significant association (*p* > 0.05) between antimicrobial resistance and the expression of virulence factors. None of the isolates showed lipase, esterase, or amylase activity, and none exhibited all the tested virulence factors simultaneously. The 11 isolates demonstrated six different virulence patterns, with Cat–Lec–Clf–Gel–Cas–Bfm–Hml (*n* = 3) being the most common (Table 5). 

## 3. Discussion

The 4.4% prevalence of *S*. *aureus* indicates a low colonization rate among donkeys in Nigeria. This low prevalence is not unexpected as it is known that *S*. *aureus* is not the predominant *Staphylococcus* species colonizing animals [28]. The recovery of the organism from the nostril of donkeys in this study is not surprising, as the nasal cavity is known to be a favorable site for staphylococcal colonization in mammals, including donkeys [29]. The detection of *S*. *aureus* and other staphylococci further underscores the potential for these donkeys to harbor diverse, potentially pathogenic, *Staphylococcus* species. The presence of *S. aureus* in the nostrils of donkeys destined for slaughter raises significant public health, occupational, and food safety concerns. Individuals in contact with these animals, such as owners, veterinarians, caretakers, butchers, and live animal market vendors, as well as consumers of donkey meat and related by-products (e.g., hides), are at risk of colonization or infection. This, in turn, increases the likelihood of indirect transmission to households and the public via contaminated persons, fomites, and the environment. Cross-contamination within slaughterhouse environments is also a concern, particularly in Nigeria, where unhygienic slaughter practices, such as the absence of personal protective equipment (PPE), are common [30]. Notably, *S. aureus* is a well-documented pathogen capable of causing various human and animal diseases, with certain strains producing lethal toxins in food products [31].

The lower prevalence of *S*. *aureus* strains (4.4%) compared to other *Staphylococcus* species (95.6%) suggests that only a small proportion of donkeys in Nigeria may be serving as potential reservoirs for *S. aureus*. It also suggests that *S. aureus* constitutes a relatively small proportion of mannitol-fermenting staphylococci in the studied donkey population. This finding is further supported by the statistically significant association between the rate of donkey colonization and *Staphylococcus* species. The 4.4% *S. aureus* colonization rate observed in this study is lower than the rates reported in Portugal (8.2%) [18], Ethiopia (13%) [21], and Tunisia (41–50%) [23,24]. Similarly, previous studies from Italy reported *S. aureus* prevalence rates of 6% in donkey milk [25] and 47.8% in conjunctival swabs [26]. In Egypt, an *S*. *aureus* prevalence rate of 50.9% in eye swabs of 56 equines, including 3 donkeys, out of 110 sampled animals, was reported [22], while studies in Nigeria documented a prevalence of 23.9-88.8% in horses [21,32]. Variations in colonization rates may be attributed to differences in isolation methods (e.g., culture media used), species detection techniques (phenotypic vs. molecular methods), sample sizes, and animal management practices across different study locations. In this study, the Staphytect® latex agglutination test was employed for the presumptive identification of *S. aureus*, with the results corroborating those obtained via MALDI-TOF analysis. The Staphytect® latex agglutination kit, which detects *S. aureus*-specific protein A and clumping factor expression, is recognized for its reliability and high specificity (100%) [33]. However, distinguishing coagulase-positive staphylococci phenotypically and serologically remains challenging. For instance, species within the *S. intermedius* group (*S. delphini, S. pseudintermedius, S. intermedius*) exhibit clumping factor variability, double zone hemolysis (like some *S*. *aureus* strains) and may agglutinate similarly to *S. aureus* in the Staphytex® kit [33,34]. Additionally, some atypical *S. aureus* strains lack coagulase or hemolytic activity, resembling non-*aureus* and non-coagulase-producing mannitol non-fermenting staphylococci [35]. In this study, none of the 11 *S. aureus* isolates that agglutinated with the Staphytex® kit exhibited double-zone hemolysis, and some were non-hemolytic (3/11), aligning with reports that *S. aureus* can be non-hemolytic [36]. Although non-*aureus* mannitol fermenters were not specifically identified, they are likely to include coagulase-positive species, such as the *S. intermedius* group (*S. delphini, S. pseudintermedius, S. intermedius*), previously reported in donkeys [24], as well as coagulase-negative mannitol-fermenting species, such as *S. haemolyticus, S. xylosus*, and *S. epidermidis*. Potential sources of *S*. *aureus* colonization in donkeys include aerosols, contaminated skin of humans or animals, environmental exposure, vector flies, food, and water.

The detection of methicillin-resistant *Staphylococcus aureus* (MRSA) in 27.3% of the isolates, suggests a moderate selection pressure against methicillin/oxacillin within the donkey population in Nigeria. However, the 1.2% prevalence of MRSA indicates that a small proportion of donkeys in the study area may serve as potential reservoirs for these resistant bacteria. This suggests that MRSA constitutes a relatively small fraction of staphylococcal colonizers in donkeys. Although the observed prevalence of methicillin resistance is low, its presence in donkeys is concerning, particularly in communities wherein these animals are used for transportation and meat production. MRS are known to be zoonotic and multidrug resistant, often conferring resistance to multiple antimicrobial classes, which can complicate treatment options, potentially resulting in increased healthcare costs and morbimortality. Notably, the MRSA isolates in this study exhibited multidrug resistance. Methicillin/oxacillin are not used in veterinary practice in Nigeria [29]. While the medical histories of the donkeys were not assessed, other β-lactam antibiotics capable of inducing methicillin resistance, such as penicillin (often combined with streptomycin), ampicillin, amoxicillin, and cephalosporins, including extended-spectrum cephalosporins, are widely used in both veterinary and human medicine in Nigeria [21,37,38]. Reports have shown that the nomads/pastoralists who keep donkeys in northern Nigeria misuse antibiotics (by using cocktails of antimicrobials for undiagnosed infections) in the management of these animals [37]. Methicillin resistance in donkeys suggests possible transmission from humans through environmental contamination (e.g., contaminated forage, water, and fomites). The acquisition of *mecA*/*mecC* genes or other resistance mechanisms may underline the observed resistance phenotype [38,39,40]. The 1.2% MRSA prevalence in this study contrasts with the findings by Gharsa et al. [23], who did not detect methicillin resistance in nasal *Staphylococcus* isolates from donkeys in Tunisia. In comparison, an MRSA prevalence of 1.1–6.3% has been reported in horses in Nigeria [21,38]. These variations may be attributed to differences in sampling techniques, detection methods (phenotypic vs. molecular), environmental factors, antimicrobial use patterns, and donkey management practices across different regions. We utilized cefoxitin (a surrogate marker for methicillin resistance) disc diffusion, as recommended by the CLSI [41], to screen for methicillin resistance. Notably, this method is recommended for assessing methicillin resistance in *S. aureus* [41]. 

Moderate-to-high resistance rates observed for gentamicin (45.5%) and penicillin (66.7%) suggest strong selection pressure for these antibiotics, aligning with previous reports on *Staphylococcus* isolates from both humans and animals in Nigeria [30,42]. Penicillin, often used in combination with aminoglycosides (e.g., streptomycin), has been extensively used in both sectors [30], likely contributing to the observed resistance patterns. Despite the absence of β-lactamase production in these isolates, penicillin resistance may be attributed to collateral methicillin resistance or other β-lactam resistance mechanisms [43]. Although the sample size of *S*. *aureus* in this study was small (11), the moderate-to-high resistance to linezolid (36.4%) and tigecycline (54.5%) suggest selective pressure against these last-resort antibiotics. This finding is particularly concerning, as these antibiotics are used as a last-resort for methicillin- and vancomycin-resistant infections and resistance to these drugs can lead to cross-resistance to multiple antimicrobial classes [2]. The WHO classifies tigecycline and linezolid under the “Reserve” group in its AWaRe (Access, Watch, Reserve) classification and recommends intensified surveillance in terms of their resistance [44]. Although tigecycline and linezolid have never been used in Nigeria’s livestock sector, their resistance may be linked to the use of related antibiotics. Tetracyclines (e.g., long-acting and short-acting oxytetracycline, doxycycline) and florfenicol, known inducers of tigecycline and linezolid resistance, respectively, are widely used in Nigeria [45,46,47]. Additionally, humans in close contact with donkeys may serve as sources of tigecycline- and linezolid-resistant organisms. Linezolid is increasingly used in human medicine in Nigeria; tetracyclines are heavily used across all sectors in the country, and tigecycline- and linezolid-resistant organisms have been reported in human communities and animal populations in the country [45,46,48]. Moreover, studies have shown that the use of non-tetracycline antimicrobials can also induce tigecycline resistance [49]. Acquired and/or non-acquired mechanisms might underline the observed tigecycline resistance [50].

Low chloramphenicol resistance (18.1%) was observed only among cefoxitin- and tigecycline-resistant isolates and may be due to cross-resistance with methicillin and/or tigecycline. However, despite being banned, chloramphenicol is still used in Nigerian livestock [36], necessitating stricter regulatory enforcement. Resistance rates reported by previous donkey studies differed from those in this study, with higher resistance to erythromycin (20%), and lower resistance to penicillin (12–18%), gentamicin (25%), and chloramphenicol (2%) [15,17]. 

This study’s multidrug resistance rate was 45.5%, suggesting moderate prior antimicrobial exposure. This is further supported by the fact that all the isolates were multiple drug resistant (resistance to two or more antibiotics) and had a moderate mean multiple antibiotic resistance index (MARI) of 0.25. An MARI greater than 0.2 indicates a high-risk source of contamination [29]. These findings emphasize the need for ongoing surveillance and prudent antimicrobial use in both human and veterinary medicine in Nigeria to mitigate resistance spread. Encouragingly, no vancomycin or inducible clindamycin resistance was detected, underscoring the importance of maintaining antimicrobial stewardship programs to preserve the efficacy of these critical antibiotics.

*S*. *aureus* is among the most common pathogens associated with hospital-acquired infections in human and veterinary settings, contributing to significant morbidity and mortality worldwide [10,11]. The expression of virulence factors by *S*. *aureus* presents a major One Health concern. In this study, all the isolates exhibited one or more tested virulence factors, demonstrating their potential to cause disease. All the isolates produced catalase (a key enzyme that protects bacteria from oxidative stress), caseinase, lecithinase, gelatinase, and clumping factor expression. While caseinase and gelatinase are proteases that facilitate bacterial dissemination by degrading proteins and contributing to biofilm formation, lecithinase lyses blood cells and enhances tissue invasion [8,23]. Other prevalent virulence factors included haemagglutinin (27.3%), which promotes adherence to host cells and colonization [8,23], and hemolysin (72.2%), which is essential for iron acquisition from host red blood cells, promoting bacterial proliferation and invasion [6]. Biofilm formation, a critical virulence factor, was detected in 54.5% of isolates, using the Congo red broth test, and in 34.6% of isolates at the air–liquid interface. The presence of biofilm-forming isolates is particularly concerning, as biofilms enhance bacterial survival in adverse environments, such as in farms and slaughterhouses, and contribute to antimicrobial resistance [29]. The identification of six distinct virulence patterns among the isolates highlights the diversity of pathogenic traits. Notably, multidrug-resistant strains, including MRSA, also exhibited these virulence factors. The combination of antimicrobial resistance and virulence traits in these isolates underscores their potential to cause severe infections in both animals and humans. 

A limitation of this study is the sample size, which may not be sufficient to accurately determine the prevalence of nasal *S*. *aureus* in donkeys in Nigeria. Additionally, the lack of molecular characterization restricted the identification of non-*S*. *aureus* species and the detection of specific antimicrobial resistance and virulence genes. Therefore, the absence of phenotypic antimicrobial resistance or virulence factors in some isolates does not exclude the possibility that they may harbor resistance genes or pathogenic traits.

## 4. Materials and Methods

### 4.1. Ethical Approval

Approval for the study was obtained from the Institutional Animal Care and Use Committee of the Faculty of Veterinary Medicine, University of Nigeria (protocol code FVM-UNN-IACUC-2023–0295 approved in February 2023). Verbal and written informed consent were secured from all the donkey transporters, owners, and caretakers, prior to sample collection.

### 4.2. Study Area

This research was conducted at the Obollo-Afor live donkey market in Udenu Local Government Area, Enugu State, southeast Nigeria (6.9153° N, 7.5139° E) [29]. The market, which is in a suboptimal hygienic condition, serves as a significant hub for the donkey trade, slaughter, and transit, particularly for donkeys transported from northern Nigeria to other parts of the country. Donkeys arriving at Obollo-Afor are temporarily housed, before continuing their journey or being processed for meat [51]. The market plays an essential role in local food security, with donkey meat constituting a substantial source of animal protein for residents of Udenu and neighboring communities. On average, 15 donkeys are slaughtered daily, with approximately 250 processed each month.

### 4.3. Sample Collection, Bacterial Isolation, and Identification

A total of 250 clinically healthy donkeys of both sexes and varying ages presented for sale, slaughter, or awaiting transportation to other states in Obollo-Afor, between May and August 2023, were sampled using a random sampling technique. A single nasal swab was collected from each animal, using a sterile swab stick. The cotton-tipped swabs were inserted approximately 6 cm into one nasal passage, ensuring contact with the nasal mucosa before withdrawal. The nasal swab samples were transported on ice packs to the Laboratory of the Department of Veterinary Microbiology and Immunology, University of Nigeria, where they were immediately processed for *Staphylococcus* isolation. Each swab was inoculated in 5 mL of tryptone soya broth, containing 6.5% NaCl for selective enrichment, and incubated at 35 ± 2 °C for 24 h under ambient conditions. A loopful of the broth culture was streaked onto mannitol salt agar (MSA) and incubated at 35 ± 2 °C for 24–48 h. Colonies with distinct morphological characteristics were observed and described accordingly. Two mannitol-fermenting colonies (yellow, medium-sized, and butyrous/glistening) were selected from each plate and purified via sub-culturing on fresh MSA, followed by incubation at 35 ± 2 °C for 24 h. The purified isolates were identified as *Staphylococcus* based on their morphological and biochemical characteristics, using Gram staining, catalase, and oxidase tests. *S*. *aureus* isolates were confirmed serologically using the *S*. *aureus* Staphytect Plus™ latex agglutination identification kit (Oxoid, Hampshire, England), according to the manufacturer’s instructions. *S*. *aureus* ATCC 25923 and *S*. *epidermidis* ATCC 12228 strains were used as positive and negative controls, respectively. The identity of the isolates that agglutinated with the Staphytect Plus™ kit and that were presumptively identified as *S*. *aureus* strains was confirmed by subjecting the isolates to matrix-assisted laser desorption/ionization time-of-flight mass spectrometry (MALDI-TOF MS), following previously described protocols [52]. A similarity log score threshold of ≥ 2.0 indicated reliable species level identification; a log score between 1.7 and 2.0 indicated presumptive species level identification and required repetition, while identifications with log scores below 1.7 were considered unreliable [52]. Isolates confirmed as *S*. *aureus* strains were sub-cultured onto nutrient agar slants, incubated at 35 ± 2 °C for 24 h, and stored in a refrigerator for a maximum of 72 h at 4 °C as stock cultures, until further analysis.

### 4.4. Antimicrobial Susceptibility Testing

Antimicrobial susceptibility testing of *S*. *aureus* isolates was performed using the disc diffusion method, following the Clinical Laboratory Standards Institute (CLSI VET01S ED7:2024) guidelines [41]. The test utilized discs (Oxoid, Hampshire, England) impregnated with seven antimicrobial agents from seven classes: β-lactams—penicillin (PEN, 10 units), aminoglycosides—gentamicin (GEN, 10 µg), oxazolidinones—linezolid (LZD, 30 µg), lincosamides—clindamycin (CLI, 2 µg), tetracyclines—doxycycline (DOX, 30 µg), macrolides—erythromycin (ERY, 15 µg), and phenicols—chloramphenicol (CHL, 30 µg). Mueller–Hinton agar (Oxoid, Hampshire, England) plates were inoculated by evenly spreading a 0.5 McFarland standard suspension of each isolate (equivalent to 1.5 × 10^8^ colony-forming units [CFUs/mL]). Within 15 m of inoculation, up to four antibiotic discs were placed at least 24 mm apart (center to center). The plates were then incubated at 35 °C ± 2 °C for 16–18 h under ambient conditions. Additionally, tigecycline (from the glycylcycline class) resistance (TIG, 15 µg) was assessed using tigecycline-impregnated discs (Oxoid, Hampshire, England) on Mueller–Hinton agar. The plates were incubated under the same conditions, and the results were interpreted according to the European Committee on Antimicrobial Susceptibility Testing (EUCAST, 2024) breakpoints for *Staphylococcus* [53]. The quality-control strain *Staphylococcus aureus* ATCC 25923 was used for susceptibility testing. Inhibition zone diameters (IZDs) were interpreted according to CLSI (VET01S ED7:2024) breakpoints for *Staphylococcus* [41], except for linezolid which followed the CLSI M100-ED35:2025 breakpoints for staphylococci [54]. The multiple antimicrobial resistance index (MARI) for each isolate was calculated using the formula *a/b*, where *a* represents the number of antibiotics to which the isolate was resistant, and *b* represents the total number of antibiotics tested [29]. Isolates resistant to at least one antimicrobial agent from three or more classes were classified as multidrug resistant (MDR) [55]. 

#### 4.4.1. Detection of Phenotypic Methicillin Resistance

Methicillin resistance in the *S*. *aureus* isolates was determined using the cefoxitin (30 µg) disc (Oxoid, Hampshire, England) diffusion method, in accordance with the CLSI guidelines [41]. In summary, a 0.5 McFarland standard suspension of the colonies was evenly spread on Mueller–Hinton agar (Oxoid, Hampshire, England), followed by the placement of a cefoxitin disc onto the plates within 15 min of inoculation. The plates were subsequently incubated at 35 °C ± 2 °C for 16–18 h under ambient conditions. Isolates with an inhibition zone diameter ≤ 21 mm around the cefoxitin disc were considered methicillin resistant [41]. *S. aureus* ATCC 25923 was used as a quality control strain.

#### 4.4.2. Detection of β-Lactamase Production

The β-lactamase production was evaluated in isolates exhibiting an inhibition zone diameter of ≥29 mm around the penicillin (10 units) disc (Oxoid, Hampshire, UK) using the penicillin zone-edge test, in accordance with the CLSI guidelines [41]. In brief, a 0.5 McFarland standard suspension of the colonies was spread onto Mueller–Hinton agar (Oxoid, Hampshire, UK), and a penicillin disc was placed onto the plates within 15 min of inoculation. The plates were then incubated at 35 °C ± 2 °C for 16–18 h under ambient conditions. Isolates displaying a sharp zone edge (a “cliff” effect) around the penicillin disc were identified as β-lactamase producers, whereas those with a diffused (“beach-like”) inhibition zone edge were classified as non-producers. *S*. *aureus* ATCC 25923 was used as the quality control strain.

#### 4.4.3. Phenotypic Vancomycin Resistance Assay

Vancomycin resistance was evaluated using the vancomycin agar screen method, in accordance with the CLSI guidelines [41]. A 10 µL aliquot of a 0.5 McFarland standard colony suspension (equivalent to 1.5 × 10^8^ colony-forming units (CFUs)/mL) was inoculated onto vancomycin agar (brain–heart infusion agar, supplemented with 4 µg/mL and 6 µg/mL vancomycin [Sigma-Aldrich, MA, USA]), which was incubated at 35 ± 2 °C for 24 h under ambient conditions. The detection of more than one colony or a light film of growth on the agar signified reduced susceptibility to vancomycin. *Enterococcus faecalis* ATCC 29212 served as the quality control strain for vancomycin susceptibility.

#### 4.4.4. Inducible Clindamycin Resistance Assay

Inducible clindamycin resistance was assessed in the *S*. *aureus* isolates using the D-zone test, following the CLSI guidelines [41]. Briefly, a 0.5 McFarland standard suspension of the colonies was spread onto Mueller–Hinton agar. Erythromycin (15 µg) and clindamycin (2 µg) discs (Oxoid, Hampshire, England) were placed 15–26 mm apart within 15 min of inoculation. The plates were then incubated at 35 °C ± 2 °C for 16–18 h under ambient conditions. The presence of a D-zone, characterized by the flattening of the inhibition zone adjacent to the erythromycin disc, indicated inducible clindamycin resistance. Meanwhile, hazy growth within the inhibition zone around the clindamycin disc, even in the absence of a D-zone, signified clindamycin resistance [41]. *S. aureus* ATCC 25923 was used as the quality control strain for inducible clindamycin resistance. 

### 4.5. Detection of Virulence Potential of S. aureus Isolates

#### 4.5.1. Catalase Activity

Catalase production was assessed using the slide catalase test [29]. A 10 µL suspension (0.5 McFarland standard) of each isolate was emulsified with 10 µL of normal saline on a slide. Immediate bubbling upon adding 20 µL of 3% hydrogen peroxide solution (Sigma-Aldrich, MA, USA) indicated catalase activity. *S. aureus* ATCC 25923 was used as a quality control strain.

#### 4.5.2. Pellicle Formation

Pellicle formation was evaluated using the broth pellicle test [56]. Colonies were inoculated into tryptic broth (Oxoid, Hampshire, England) and incubated stagnant at 35 ± 2 °C for 24 h. An aggregative matrix layer on the broth surface indicated pellicle formation. *S. aureus* ATCC 6538 served as a control strain.

#### 4.5.3. Biofilm Production

Biofilm formation was assessed using the Congo red broth test [57]. A 10 µL suspension (0.5 McFarland standard) of each isolate was inoculated into Congo red broth (tryptic broth (Oxoid, Hampshire, UK), with 0.3% wv^−1^ sucrose (Sigma-Aldrich, MA, USA)) and 0.08% vv^−1^ Congo red dye (Sigma-Aldrich, MA, USA)), and incubated at 35 ± 2°C for 48 h under ambient conditions. Blackened broth indicated biofilm production, while yellow, red, brown, or black–red broth suggested negative results. *S. aureus* ATCC 6538 was used as a positive control.

#### 4.5.4. Hemolysin Production

Hemolysin production was determined using the plate hemolysis test [30]. A 10 µL suspension (0.5 McFarland standard) was spotted onto blood agar (tryptic agar (Oxoid, Hampshire, UK) with 5% vv^−1^ sheep blood) and incubated at 35 ± 2 °C for 24 h in ambient air. Transparent or greenish zones around colonies indicated hemolysis [29]. *S. aureus* ATCC 25923 served as the positive control.

#### 4.5.5. Hemagglutinin Expression

Hemagglutinin production was evaluated using the hemagglutination test [58]. A 25 µL suspension of each isolate in tryptic soy broth (Oxoid, Hampshire, UK) was mixed with 3% chicken red blood cells on a tile, rocked for 5 min, and examined for cell clumping. *S. aureus* ATCC 25923 was used as the positive control.

#### 4.5.6. Gelatinase Activity

Gelatinase production was assessed using the gelatin liquefaction test [30]. A 10 µL suspension (0.5 McFarland standard) was spotted onto gelatin agar (trypticase with 3% wv^−1^ gelatin (Sigma-Aldrich, MA, USA)) and incubated at 35 ± 2 °C for 48 h, aerobically. A transparent halo after flooding with Frazier solution, which contained 15 g mercuric chloride, 20 mL 37% hydrochloric acid, and100 mL distilled water, was considered indicative of gelatinase activity [30]. *S. aureus* ATCC 25923 was used as a positive control.

#### 4.5.7. Caseinase Activity

Caseinase production was established using the casein hydrolysis test [30]. A 10 µL suspension (0.5 McFarland standard) was spotted onto casein agar (trypticase with 15% wv^−1^ soluble casein (Sigma-Aldrich, MA, USA)) and incubated at 35 ± 2 °C for 24 h under ambient conditions. Clear zones surrounding growth after flooding with the Frazier solution indicated caseinase production [29]. *S. aureus* ATCC 25923 was used as a positive control.

#### 4.5.8. Lipase and Esterase Activity

Lipase and esterase production were evaluated as per the method outlined by Ramnath et al. [59]. A 10 µL suspension (0.5 McFarland standard) was spotted onto tween 80 (trypticase with 1% vv^−1^ tween 80 (Sigma-Aldrich, MA, USA)) and tween 20 (tryptone soya agar with 1% vv^−1^ tween 20 (Sigma-Aldrich, MA, USA)) agar plates. The plates were incubated at 35 ± 2 °C for 48 h in ambient air. Yellowish zones indicated lipase and esterase production [59]. *S. aureus* ATCC 25923 was used as a positive control.

#### 4.5.9. Amylase Activity

Amylase production was assessed using starch hydrolysis [60,61]. A 10 µL suspension (0.5 McFarland standard) was spotted onto starch agar (trypticase with 1% wv^−1^ soluble starch (Sigma-Aldrich, MA, USA)) and incubated at 35 ± 2 °C for 48 h aerobically. Brownish black halos after flooding with Lugol’s iodine indicated amylase activity [60,61]. *S. epidermidis* ATCC 14990 was used as a positive control.

### 4.6. Data Analysis

The test results were recorded in Microsoft Excel™ (Microsoft, Redmond, WA, USA). The data on the occurrence of *S*. *aureus*, antimicrobial resistance, and virulence potential were then exported to SPSS v.15.0 (SPSS, Chicago, IL, USA) for analysis. The mean multiple antibiotic resistance index (MARI) was calculated by summing up the MARI values of all the isolates and dividing by the total number of isolates. The frequency, percentage, and 95% confidence intervals were determined. The associations between variables (resistance and virulence) and the isolated *Staphylococcus* strains were assessed using the chi-squared (χ^2^) test or Fisher’s exact test.

## 5. Conclusions

This study shows that although a small proportion of donkeys in Nigeria may be serving as reservoirs of *S*. *aureus*, these donkeys can directly harbor antimicrobial-resistant staphylococci, including methicillin-resistant *S*. *aureus* strains. Thus, their presence poses a serious public health concern. These pathogens increase the risk of disease outbreaks and may complicate antimicrobial treatment in affected individuals. Transmission to humans can occur through direct contact or consumption of contaminated meat, further exacerbating the spread of resistance. The proliferation of multidrug-resistant *S. aureus* could have a profound impact on the epidemiology of antibiotic resistance in Nigeria. Given the global significance of *S. aureus* as a leading antimicrobial-resistant pathogen, it is essential to strengthen hygiene practices among donkey handlers and implement stricter regulations on antibiotic use in veterinary medicine in Nigeria.

## Figures and Tables

**Table 1:** Prevalence of *Staphylococcus aureus* in donkeys from Obollo-Afor, southeast Nigeria.

Organism	Number of Positive Samples (*n* = 250)	Percentage	95% Confidence Interval	Odds Ratio
*S*. *aureus*	11	4.4	1.86–6.94	0.0021
Other *Staphylococcus* species	239	95.6	93.06–98.14	0.0021

*n* = number of samples, S = *Staphylococcus*.

**Table 2:** Antimicrobial susceptibility profile of *Staphylococcus aureus* from donkeys in Obollo-Afor, southeast Nigeria.

Antimicrobial Class	Antimicrobial Agent	Number (%)		*p*-Value
		Resistant	Susceptible	
β-lactams	Penicillin	8 (66.7)	4 (33.3)	0.1984
	Cefoxitin	3 (27.3)	8 (72.7)	0.0861
Tetracyclines	Doxycycline	0 (0.0)	11 (100)	0.00000284 *
	Tigecycline	6 (54.5)	6 (45.5)	1.0000
Glycopeptides	Vancomycin	0 (0.0)	0 (0.0)	NA
Oxazolidinones	Linezolid	4 (36.4)	7 (63.6)	0.3949
Lincosamides	Clindamycin	0 (0.0)	0 (0.0)	NA
Phenicols	Chloramphenicol	2 (18.1)	9 (81.9)	0.0089 *
Aminoglycosides	Gentamicin	5 (45.5)	6 (54.5)	1.0000
Macrolides	Erythromycin	0 (0.0)	0 (0.0)	NA

* Significant difference at *p* < 0.05; NA = not applicable.

**Table 3:** Resistance to antimicrobial agents among *Staphylococcus aureus* from donkeys.

Number of Antimicrobial Agents	Number (%) of Resistant Isolates (*n* = 11)
0	0 (0.0)
1	0 (0.0)
2	6 (54.5)
3	2 (18.2)
4	2 (18.2)
5	1 (9.1)

*n* = number of isolates.

**Table 4:** Antimicrobial resistance patterns and multiple antimicrobial resistance indices of *Staphylococcus aureus* from donkeys.

SN	No. of Antimicrobials (MARIs)	Antimicrobial Resistance Pattern	Total (*n* = 11)	No. of Antimicrobial Classes	No. (%) of MDR Strains
1	2 (0.2)	TIG–GEN	1	2	5 (45.5)
2		LZD–GEN	1		
3		PEN–LZD	3		
4		PEN–TIG	1		
5	3 (0.3)	PEN–TIG–CHL	2	3	
6	4 (0.4)	PEN–FOX–TIG–GEN	2		
7	5 (0.5)	PEN–FOX–TIG–GEN–CHL	1	4	

*n* = number of isolates, S. = *Staphylococcus*, MDR = multidrug-resistant, MARI = multiple antimicrobial resistance index, FOX = cefoxitin, PEN = penicillin, GEN = gentamicin, TIG = tigecycline, LZD = linezolid, CHL = chloramphenicol.

**Table 5:** Phenotypic virulence patterns of *Staphylococcus aureus* from donkeys in Obollo-Afor, southeast Nigeria.

S/N	Virulence Pattern	Number of Isolates	% Frequency (*n* = 11)
1	Cat–Lec–Clf–Gel–Cas–Bfm–Hgl–Hml	2	18.2
2	Cat–Lec–Clf–Gel–Cas–Hml	2	18.2
3	Cat–Lec–Clf–Gel–Cas–Hgl–Pel–Hml	1	9.1
4	Cat–Lec–Clf–Gel–Cas–Pel	2	18.2
5	Cat–Lec–Clf–Gel–Cas–Bfm–Pel	1	9.1
6	Cat–Lec–Clf–Gel–Cas–Bfm–Hml	3	27.3

Cat = catalase, Lec = lecithinase, Clf = clumping factor, Bfm = biofilm, Hgl = hemagglutinin, Gel = gelatinase, Cas = caseinase, Pel = pellicle, Hml = hemolysin.

## Data Availability

The original contributions presented in this study are included in the article. Further inquiries can be directed to the corresponding author.

## References

[1] Bertelloni F., Cagnoli G., Ebani V.V. (2021). Virulence and Antimicrobial Resistance in Canine *Staphylococcus* spp. Isolates. Microorg..

[2] Esposito S., Blasi F., Curtis N., Kaplan S., Lazzarotto T., Meschiari M., Mussini C., Peghin M., Rodrigo C., Vena A. (2023). New Antibiotics for *Staphylococcus aureus* Infection: An Update from the World Association of Infectious Diseases and Immunological Disorders (WAidid) and the Italian Society of Anti-Infective Therapy (SITA). Antibiotics.

[3] Derdak R., Quinteiro J., Sakoui S., Addoum B., Rodríguez Castro J., Rey Méndez M., Soukri A., El Khalfi B. (2021). Isolation and Identification of Dominant Bacteria from Raw Donkey Milk Produced in a Region of Morocco by QIIME 2 and Evaluation of Their Antibacterial Activity. Sci. World J..

[4] Ahmad-Mansour N., Loubet P., Pouget C., Dunyach-Remy C., Sotto A., Lavigne J.P., Molle V. (2021). *Staphylococcus aureus* Toxins: An Update on Their Pathogenic Properties and Potential Treatments. Toxins.

[5] Tam K., Torres V.J. (2019). *Staphylococcus aureus* Secreted Toxins and Extracellular Enzymes. Microbiol. Spectr..

[6] Pillay S., Zishiri O.T., Adeleke M.A. (2018). Prevalence of Virulence Genes in *Enterococcus* Species Isolated from Companion Animals and Livestock. Onderstepoort J. Vet. Res..

[7] Mohsin S., Amin M.N. (2023). Superbugs: A Constraint to Achieving the Sustainable Development Goals. Bull. Natl. Res. Cent..

[8] Mohsen N., Vollset S.E., Ikuta K.S. (2024). Global Burden of Bacterial Antimicrobial Resistance 1990–2021: A Systematic Analysis with Forecasts to 2050. Lancet.

[9] Murray C.J., Ikuta K.S., Sharara F., Swetschinski L., Robles Aguilar G., Gray A., Han C., Bisignano C., Rao P., Wool E. (2022). Global Burden of Bacterial Antimicrobial Resistance in 2019: A Systematic Analysis. Lancet.

[10] Ikuta K.S., Swetschinski L.R., Robles Aguilar G., Sharara F., Mestrovic T., Gray A.P., Davis Weaver N., Wool E.E., Han C., Gershberg Hayoon A. (2022). Global Mortality Associated with 33 Bacterial Pathogens in 2019: A Systematic Analysis for the Global Burden of Disease Study 2019. Lancet.

[11] De Oliveira D.M.P., Forde B.M., Kidd T.J., Harris P.N.A., Schembri M.A., Beatson S.A., Paterson D.L., Walker M.J. (2020). Antimicrobial Resistance in ESKAPE Pathogens. Clin. Microbiol. Rev..

[12] Aloke C., Achilonu I. (2023). Coping with the ESKAPE Pathogens: Evolving Strategies, Challenges and Future Prospects. Microb. Pathog..

[13] OPS Group (2024). WHO Bacterial Priority Pathogens List, 2024: Bacterial Pathogens of Public Health Importance to Guide Research, Development and Strategies to Prevent and Control Antimicrobial Resistance.

[14] Mills G. (2022). Assessing Antimicrobial Use and Practices in Equids. Vet. Rec..

[15] Norris S.L., Little H.A., Ryding J., Raw Z. (2021). Global Donkey and Mule Populations: Figures and Trends. PLoS ONE.

[16] Hassan M.R., Steenstra F.A., Udo H.M.J. (2013). Benefits of Donkeys in Rural and Urban Areas in Northwest Nigeria. Afr. J. Agric. Res..

[17] Maigari M.A., Dantani U., Yelwa M.M., Ibrahim A. (2020). Scavenging for Ejiao’s Raw Material and the Extinction of Donkeys in Nigeria. Glob. J. Sociol. Curr. Issues.

[18] Goodrum F., Theuri S., Mutua E., Carder G. (2022). The Donkey Skin Trade: Challenges and Opportunities for Policy Change. Glob. Policy.

[19] Silva V., Alfarela C., Caniça M., Manageiro V., Nóvoa M., Leiva B., Kress M., Capelo J.L., Poeta P., Igrejas G. (2022). A One Health Approach Molecular Analysis of *Staphylococcus aureus* Reveals Distinct Lineages in Isolates from Miranda Donkeys (*Equus asinus*) and Their Handlers. Antibiotics.

[20] Ituma O.E. (2014). Acceptability and Consumption of Donkey Meat in Ebonyi State. Glob. J. Bio-Sci. Technol..

[21] Jassim S.A., Kandala N.J. (2021). Molecular Detection of Enterotoxin Genes of Multiresistant *Staphylococcus aureus* Isolates from Different Sources of Food. Iraqi J. Sci..

[22] Debelu T., Aklilu N., Sisay T., Desissa F. (2014). Isolation and Identification of Aerobic Bacterial Flora from the Upper Respiratory Tract of Donkeys in Central Ethiopia. J. Veterainry Med. Anim. Health.

[23] Tahoun A., Elnafarawy H.K., El-Sharkawy H., Rizk A.M., Alorabi M., El-Shehawi A.M., Youssef M.A., Ibrahim H.M.M., El-Khodery S. (2022). The Prevalence and Molecular Biology of *Staphylococcus aureus* Isolated from Healthy and Diseased Equine Eyes in Egypt. Antibiotics.

[24] Gharsa H., Ben Sallem R., Ben Slama K., Gómez-Sanz E., Lozano C., Jouini A., Klibi N., Zarazaga M., Boudabous A., Torres C. (2012). High Diversity of Genetic Lineages and Virulence Genes. in Nasal *Staphylococcus aureus* Isolates from Donkeys Destined to Food Consumption in Tunisia with Predominance of the Ruminant Associated CC133 Lineage. BMC Vet. Res..

[25] Gharsa H., Slama K.B., Gómez-Sanz E., Gómez P., Klibi N., Zarazaga M., Boudabous A., Torres C. (2015). Characterisation of Nasal *Staphylococcus delphini* and *Staphylococcus pseudintermedius* Isolates from Healthy Donkeys in Tunisia. Equine Vet. J..

[26] Pilla R., Daprà V., Zecconi A., Piccinini R. (2010). Hygienic and Health Characteristics of Donkey Milk during a Follow-up Study. J. Dairy. Res..

[27] Foti M., Fisichella V., Giacopello C. (2013). Detection of Methicillin-Resistant Staphylococcus Aureus (MRSA) in the Microbial Flora from the Conjunctiva of Healthy Donkeys from Sicily (Italy). Vet. Ophthalmol..

[28] Little S.V., Hillhouse A.E., Lawhon S.D., Bryan L.K., Fey P.D. (2021). Analysis of Virulence and Antimicrobial Resistance Gene Carriage in *Staphylococcus aureus* Infections in Equids Using Whole-Genome Sequencing. Msphere.

[29] Thomson P., García P., Miles J., Isla D., Yáñez C., Santibáñez R., Núñez A., Flores-Yáñez C., Del Río C., Cuadra F. (2022). Isolation and Identification of *Staphylococcus* Species Obtained from Healthy Companion Animals and Humans. Vet. Sci..

[30] Nwobi O.C., Anyanwu M.U., Jaja I.F., Nwankwo I.O., Okolo C.C., Nwobi C.A., Ezenduka E.V., Oguttu J.W. (2023). *Staphylococcus aureus* in Horses in Nigeria: Occurrence, Antimicrobial, Methicillin and Heavy Metal Resistance and Virulence Potentials. Antibiotics.

[31] Argudín M.Á., Mendoza M.C., Rodicio M.R. (2010). Food Poisoning and *Staphylococcus aureus* Enterotoxins. Toxins.

[32] Sanda M.I., Idris A. (2021). M Nasopharyngeal Carriage of *Staphylococcus aureus* among Horses and Horse Handlers in Kano Metropolis, Nigeria. UMYU J. Microbiol. Res. (UJMR).

[33] Shah P., Sah R., Sharma A., Khanal B., Bhattarai N.R. (2023). Evaluation of Latex Agglutination Test for Rapid Identification of *Staphylococcus aureus* Isolated from Pyogenic Wound Infections at a Tertiary Care Hospital. Kathmandu Univ. Med. J..

[34] Lainhart W., Yarbrough M.L., Burnham C.A.D. (2018). The Brief Case: *Staphylococcus* Intermedius Group-Look What the Dog Dragged In. J. Clin. Microbiol..

[35] Latorre-Fernández J., Aspiroz C., Abdullahi I.N., Campaña-Burguet A., Eguizábal P., González-Azcona C., Tenorio C., Zarazaga M., Shittu A.O., Lozano C. (2025). Evaluation of the Double-Zone Hemolysis (DZH) Test for the Detection of Livestock-Associated Methicillin-Resistant *Staphylococcus aureus*. Microbiol. Spectr..

[36] Thakur P., Nayyar C., Tak V., Saigal K. (2017). Mannitol-Fermenting and Tube Coagulase-Negative Staphylococcal Isolates: Unraveling the Diagnostic Dilemma. J. Lab. Physicians.

[37] Adesokan H.K., Akanbi I.O., Akanbi I.M., Obaweda R.A. (2015). Pattern of Antimicrobial Usage in Livestock Animals in South-Western Nigeria: The Need for Alternative Plans. Onderstepoort J. Vet. Res..

[38] Alhaji N.B., Isola T.O. (2018). Antimicrobial Usage by Pastoralists in Food Animals in North-Central Nigeria: The Associated Socio-Cultural Drivers for Antimicrobials Misuse and Public Health Implications. One Health.

[39] Ali T., Basit A., Karim A.M., Lee J.H., Jeon J.H., Rehman S.U., Lee S.H. (2021). Mutation-Based Antibiotic Resistance Mechanism in Methicillin-Resistant *Staphylococcus aureus* Clinical Isolates. Pharmaceuticals.

[40] Peacock S.J., Paterson G.K. (2015). Mechanisms of Methicillin Resistance in *Staphylococcus aureus*. Annu. Rev. Biochem..

[41] (2024). Performance Standards for Antimicrobial Disk and Dilution Susceptibility Tests for Bacteria Isolated from Animals.

[42] Ezeh C.K., Eze C.N., Dibua M.E.U., Emencheta S.C. (2023). A Meta-Analysis on the Prevalence of Resistance of *Staphylococcus aureus* to Different Antibiotics in Nigeria. Antimicrob. Resist. Infect. Control.

[43] Fisher J.F., Mobashery S. (2016). β-Lactam Resistance Mechanisms: Gram-Positive Bacteria and Mycobacterium Tuberculosis. Cold Spring Harb. Perspect. Med..

[44] Zanichelli V., Sharland M., Cappello B., Moja L., Getahun H., Pessoa-Silva C., Sati H., van Weezenbeek C., Balkhy H., Simão M. (2023). The WHO AWaRe (Access, Watch, Reserve) Antibiotic Book and Prevention of Antimicrobial Resistance. Bull. World Health Organ..

[45] Ngbede E.O., Sy I., Akwuobu C.A., Nanven M.A., Adikwu A.A., Abba P.O., Adah M.I., Becker S.L. (2023). Carriage of Linezolid-Resistant Enterococci (LRE) among Humans and Animals in Nigeria: Coexistence of the Cfr, OptrA, and PoxtA Genes in Enterococcus Faecium of Animal Origin. J. Glob. Antimicrob. Resist..

[46] Anyanwu M.U., Ikenna-Ezeh N.H., Okafor S.C., Ezemuoka C.F., Nwobi O.C., Ogunniran T.M., Obodoechi L.O., Okorie-Kanu O.J., Mgbeahuruike A.C., Okosi I.R. (2024). Commercial Day-Old Chicks in Nigeria Are Potential Reservoirs of Colistin- and Tigecycline-Resistant Potentially Pathogenic *Escherichia coli*. Antibiotics.

[47] Adesoji A.T., Call D.R. (2020). Molecular Analysis of Florfenicol-Resistant Bacteria Isolated from Drinking Water Distribution Systems in Southwestern Nigeria. J. Glob. Antimicrob. Resist..

[48] Abdu A., Lamikanra A. (2016). Linezolid and Methicillin Resistances in *S. aureus* Isolated from the Anterior Nares of Apparently Healthy Undergraduates of the Niger Delta University, Nigeria. Br. Microbiol. Res. J..

[49] Lv L., Wan M., Wang C., Gao X., Yang Q., Partridge S.R., Wang Y., Zong Z., Doi Y., Shen J. (2020). Emergence of a Plasmid-Encoded Resistance-Nodulation- Division Efflux Pump Conferring Resistance to Multiple Drugs, Including Tigecycline, in *Klebsiella pneumoniae*. mBio.

[50] Heidary M., Sholeh M., Koupaei M., Asadi A., Khah S.M., Kheirabadi F., Saeidi P., Darbandi A., Taheri B., Ghanavati R. (2024). Prevalence of Tigecycline Resistance in Methicillin-Resistant *Staphylococcus aureus*: A Systematic Review and Meta-Analysis. Diagn. Microbiol. Infect. Dis..

[51] Okoroafor O.N., Aneru E., Eze J.I., Chukwudi I.C., Anagor T., Kazeem H., Ngene A.A. (2020). Prevalence of Mycotic Agents Isolated from Skin Lesions of Trade Horses in Obollor-Afor, Enugu State, Nigeria. Sokoto J. Vet. Sci..

[52] Rosa N.M., Penati M., Fusar-Poli S., Addis M.F., Tola S. (2022). Species Identification by MALDI-TOF MS and Gap PCR-RFLP of Non-Aureus *Staphylococcus*, *Mammaliicoccus*, and *Streptococcus* spp. Associated with Sheep and Goat Mastitis. Vet. Res..

[53] EUCAST The European Committee on Antimicrobial Susceptibility Testing (2024). Breakpoint Tables for Interpretation of MICs and Zone Diameters. Version 14.0. http://www.Eucast.Org.

[54] (2025). Performance Standards for Antimicrobial Susceptibility Testing.

[55] Magiorakos A.P., Srinivasan A., Carey R.B., Carmeli Y., Falagas M.E., Giske C.G., Harbarth S., Hindler J.F., Kahlmeter G., Olsson-Liljequist B. (2012). Multidrug-Resistant, Extensively Drug-Resistant and Pandrug-Resistant Bacteria: An International Expert Proposal for Interim Standard Definitions for Acquired Resistance. Clin. Microbiol. Infect..

[56] Dawadi P., Khanal S., Prasai Joshi T., KC S., Tuladhar R., Maharjan B.L., Darai A., Joshi D.R. (2022). Antibiotic Resistance, Biofilm Formation and Sub-Inhibitory Hydrogen Peroxide Stimulation in Uropathogenic *Escherichia coli*. Microbiol. Insights.

[57] Lee J.S., Bae Y.M., Han A., Lee S.Y. (2016). Development of Congo Red Broth Method for the Detection of Biofilm-Forming or Slime-Producing *Staphylococcus* sp. LWT.

[58] Hagos D.G., Mezgebo T.A., Berhane S., Medhanyie A.A. (2019). Biofilm and Hemagglutinin Formation: A Hallmark for Drug Resistant Uropathogenic *Escherichia coli*. BMC Res. Notes.

[59] Ramnath L., Sithole B., Govinden R. (2017). Identification of Lipolytic Enzymes Isolated from Bacteria Indigenous to *Eucalyptus* Wood Species for Application in the Pulping Industry. Biotechnol. Rep..

[60] Al-Dhabi N.A., Esmail G.A., Ghilan A.K.M., Arasu M.V., Duraipandiyan V., Ponmurugan K. (2020). Isolation and Purification of Starch Hydrolysing Amylase from *Streptomyces* sp. Al-Dhabi-46 Obtained from the Jazan Region of Saudi Arabia with Industrial Applications. J. King Saud. Univ. Sci..

[61] Preda M., Mihai M.M., Popa L.I., Diţu L.M., Holban A.M., Manolescu L.S.C., Popa G.L., Muntean A.A., Gheorghe I., Chifiriuc C.M. (2021). Phenotypic and Genotypic Virulence Features of Staphylococcal Strains Isolated from Difficult-to-Treat Skin and Soft Tissue Infections. PLoS ONE.

